# Address

**Published:** 1846-12

**Authors:** Eleazar Parmly


### ARTICLE V.

*Addressed to the Members of the American Society of Dental Surgeons, at their Seventh Annual Meeting*, held in the Hall of the Medical Department of the University of New York, August 4th, 1846. By Eleazar Parmly, M. D., D. D. S.

Welcome, cherished friends, once more,
To this highly favored hall,
Where the light of Science beams,
Welcome one, and welcome all.

Here the highest gifts are held,
Nature's myst'ries making plain,
By a Pattison, Revere,
Bedford, Draper, Mott and Paine ;*

Names well known in all the world,
Honored, too, in ev'ry clime,
And will, on the page of fame,
Brightly shine through coming time.

Then let ardor fill each breast,
Let our zeal and love be shown,
By imparting to our cause,
That which truth may proudly own.

Let our efforts all be made,
Here to gain a rank and place,
Where our cause may safely rest,
Shielding it from past disgrace.

And, united, we've the power
To restore its fading name,
And to seasons not remote,
Bring it to distinguished fame.

* The Faculty of the College.

But if strife should intervene,
  With'ring in its very dawn,
All our efforts will be lost,
  All our labors will be gone.

Gone, but not forgotten; No!
  Hate and envy both will tell,
Of the height on which we stood,
  Of the depth to which we fell.

Let our thoughts and hopes ascend,
  From the darkly flowing flood,
Where the poison'd Hydra lives,
  Blighting all that's pure and good;

To that proud and lofty height,
  Where ambition loudly calls,
Where the sun of Science gilds
  Ev'ry thing on which it falls.

Let this be our noble aim,
  Thither let our efforts tend,
Science then will own our cause,
  Science then will be our friend;

To record, when we are gone,
  What our efforts here have done;
What that cause for truth has gained,
  What for DENTOLOGIA won.

